# Supplementary material for: A systematic survey of regional multi-taxon biodiversity: evaluating strategies and coverage
Source: BMC Ecol. 2019 Oct 15;19:43. doi: 10.1186/s12898-019-0260-x (PMC6792264; doi:10.1186/s12898-019-0260-x)
Supplement: Supplementary file 1 — Additional file 1: Appendix A. Site characteristics for each of the 130 40 × 40 m sites. [file 12898_2019_260_MOESM1_ESM.docx]

**Appendix**

**Appendix A:** Table holding the information about class and UTM coordinates for each of the 130 sites included in the survey. The natural sites (n = 90) were selected to cover three gradients (successional stage, moisture, fertility) and the class for these were the combination of the *a priori* assumed levels of the three gradients successional stage, moisture, and fertility. The sites representing the cultivated landscape (n =30) were grouped in six classes of which three were plantations (beech, oak, and spruce) and three were arable (rotational, oldfield, ley), and finally 10 sites were assigned to the class perceived areas of high species richness (highspcrich). The table also provides the site ID, assumed successional stage (early, mid, late), assumed moisture level (dry, moist, wet), and assumed fertility level (poor, rich) for the natural sites as well as the coordinates (UTMX and UTMY).

| Site ID | | Class | | Successional stage | | Moisture | | Fertility | | UTMX | | UTMY | |
| --- | --- | --- | --- | --- | --- | --- | --- | --- | --- | --- | --- | --- | --- |
| 1 | EarlyDryPoor | | Early | | Dry | | Poor | | 581439 | | 6388139 | |  |
| 2 | EarlyWetRich | | Early | | Moist | | Rich | | 564449 | | 6383379 | |  |
| 3 | EarlyWetPoor | | Early | | Wet | | Poor | | 583805 | | 6390025 | |  |
| 4 | Rotational | | - | | - | | - | | 555479 | | 6375310 | |  |
| 5 | HighSpcRich | | - | | - | | - | | 552708 | | 6375363 | |  |
| 6 | Ley | | - | | - | | - | | 564600 | | 6382577 | |  |
| 7 | MidMoistRich | | Mid | | Moist | | Rich | | 563198 | | 6383594 | |  |
| 8 | MidWetPoor | | Mid | | Wet | | Poor | | 583990 | | 6389854 | |  |
| 9 | Spruce | | - | | - | | - | | 574170 | | 6384473 | |  |
| 10 | EarlyDryRich | | Early | | Dry | | Rich | | 479150 | | 6330451 | |  |
| 11 | EarlyMoistPoor | | Early | | Moist | | Poor | | 497046 | | 6333482 | |  |
| 12 | LateMoistPoor | | Late | | Moist | | Poor | | 473347 | | 6321637 | |  |
| 13 | LateWetPoor | | Late | | Wet | | Poor | | 495753 | | 6323436 | |  |
| 14 | MidDryPoor | | Mid | | Dry | | Poor | | 480429 | | 6330041 | |  |
| 15 | MidMoistPoor | | Mid | | Moist | | Poor | | 478318 | | 6324607 | |  |
| 16 | Oldfield | | - | | - | | - | | 494962 | | 6323037 | |  |
| 17 | Oak | | - | | - | | - | | 494653 | | 6322392 | |  |
| 18 | EarlyMoistRich | | Early | | Moist | | Rich | | 492405 | | 6322388 | |  |
| 19 | HighSpcRich | | - | | - | | - | | 552576 | | 6301441 | |  |
| 20 | LateDryPoor | | Late | | Dry | | Poor | | 551028 | | 6297143 | |  |
| 21 | LateDryRich | | Late | | Dry | | Rich | | 556187 | | 6305594 | |  |
| 22 | LateMoistRich | | Late | | Moist | | Rich | | 574000 | | 6311083 | |  |
| 23 | LateWetRich | | Late | | Wet | | Rich | | 574846 | | 6310504 | |  |
| 24 | MidDryRich | | Mid | | Dry | | Rich | | 548846 | | 6298056 | |  |
| 25 | MidWetRich | | Mid | | Wet | | Rich | | 550047 | | 6299093 | |  |
| 26 | Beech | | - | | - | | - | | 551954 | | 6296985 | |  |
| 27 | EarlyDryPoor | | Early | | Dry | | Poor | | 446101 | | 6241311 | |  |
| 28 | EarlyWetPoor | | Early | | Wet | | Poor | | 461377 | | 6224901 | |  |
| 29 | Rotational | | - | | - | | - | | 450191 | | 6239198 | |  |
| 30 | MidDryPoor | | Mid | | Dry | | Poor | | 469509 | | 6233884 | |  |
| 31 | MidMoistRich | | Mid | | Moist | | Rich | | 462049 | | 6224867 | |  |
| 32 | MidWetPoor | | Mid | | Wet | | Poor | | 461031 | | 6225250 | |  |
| 33 | MidWetRich | | Mid | | Wet | | Rich | | 448189 | | 6241792 | |  |
| 34 | Oldfield | | - | | - | | - | | 446448 | | 6231765 | |  |
| 35 | Beech | | - | | - | | - | | 468639 | | 6233492 | |  |
| 36 | EarlyMoistRich | | Early | | Moist | | Rich | | 448267 | | 6186362 | |  |
| 37 | EarlyWetRich | | Early | | Wet | | Rich | | 448741 | | 6181179 | |  |
| 38 | HighSpcRich | | - | | - | | - | | 452316 | | 6170040 | |  |
| 39 | LateMoistPoor | | Late | | Moist | | Poor | | 449133 | | 6172346 | |  |
| 40 | Ley | | - | | - | | - | | 450647 | | 6183553 | |  |
| 41 | MidDryRich | | Mid | | Dry | | Rich | | 441993 | | 6157820 | |  |
| 42 | MidMoistPoor | | Mid | | Moist | | Poor | | 447794 | | 6171266 | |  |
| 43 | Oak | | - | | - | | - | | 450717 | | 6169923 | |  |
| 44 | EarlyDryRich | | Early | | Dry | | Rich | | 493481 | | 6110733 | |  |
| 45 | EarlyMoistPoor | | Early | | Moist | | Poor | | 495874 | | 6114067 | |  |
| 46 | HighSpcRich | | - | | - | | - | | 497380 | | 6096051 | |  |
| 47 | LateDryPoor | | Late | | Dry | | Poor | | 496882 | | 6117838 | |  |
| 48 | LateDryRich | | Late | | Dry | | Rich | | 503771 | | 6127399 | |  |
| 49 | LateMoistRich | | Late | | Moist | | Rich | | 498339 | | 6096492 | |  |
| 50 | LateWetPoor | | Late | | Wet | | Poor | | 499012 | | 6096769 | |  |
| 51 | LateWetRich | | Late | | Wet | | Rich | | 504292 | | 6127348 | |  |
| 52 | Spruce | | - | | - | | - | | 496808 | | 6115356 | |  |
| 53 | EarlyDryRich | | Early | | Dry | | Rich | | 595014 | | 6228997 | |  |
| 54 | Rotational | | - | | - | | - | | 591617 | | 6239515 | |  |
| 55 | HighSpcRich | | - | | - | | - | | 614408 | | 6240840 | |  |
| 56 | LateDryRich | | Late | | Dry | | Rich | | 591098 | | 6239031 | |  |
| 57 | MidDryPoor | | Mid | | Dry | | Poor | | 594494 | | 6230173 | |  |
| 58 | MidDryRich | | Mid | | Dry | | Rich | | 595138 | | 6218271 | |  |
| 59 | MidMoistPoor | | Mid | | Moist | | Poor | | 594188 | | 6217924 | |  |
| 60 | MidMoistRich | | Mid | | Moist | | Rich | | 598418 | | 6231974 | |  |
| 61 | EarlyWetPoor | | Early | | Wet | | Poor | | 596888 | | 6232144 | |  |
| 62 | EarlyDryPoor | | Early | | Dry | | Poor | | 525829 | | 6215685 | |  |
| 63 | MidWetPoor | | Mid | | Wet | | Poor | | 522324 | | 6217641 | |  |
| 64 | EarlyMoistRich | | Early | | Moist | | Rich | | 543873 | | 6212230 | |  |
| 65 | EarlyMoistPoor | | Early | | Moist | | Poor | | 524781 | | 6215998 | |  |
| 66 | EarlyWetRich | | Early | | Wet | | Rich | | 545568 | | 6213415 | |  |
| 67 | HighSpcRich | | - | | - | | - | | 539737 | | 6214622 | |  |
| 68 | LateDryPoor | | Late | | Dry | | Poor | | 538527 | | 6224868 | |  |
| 69 | LateMoistPoor | | Late | | Moist | | Poor | | 532798 | | 6221134 | |  |
| 70 | LateWetPoor | | Late | | Wet | | Poor | | 530849 | | 6220234 | |  |
| 71 | LateMoistRich | | Late | | Moist | | Rich | | 553506 | | 6170326 | |  |
| 72 | LateWetRich | | Late | | Wet | | Rich | | 545923 | | 6172033 | |  |
| 73 | Ley | | - | | - | | - | | 543418 | | 6162941 | |  |
| 74 | MidWetRich | | Mid | | Wet | | Rich | | 543671 | | 6162515 | |  |
| 75 | Oldfield | | - | | - | | - | | 534312 | | 6169169 | |  |
| 76 | Beech | | - | | - | | - | | 553916 | | 6171233 | |  |
| 77 | Spruce | | - | | - | | - | | 554670 | | 6171805 | |  |
| 78 | Oak | | - | | - | | - | | 547299 | | 6171972 | |  |
| 79 | EarlyDryPoor | | Early | | Dry | | Poor | | 686535 | | 6212641 | |  |
| 80 | HighSpcRich | | - | | - | | - | | 686452 | | 6212178 | |  |
| 81 | LateDryPoor | | Late | | Dry | | Poor | | 704258 | | 6206120 | |  |
| 82 | LateMoistRich | | Late | | Moist | | Rich | | 704170 | | 6207381 | |  |
| 83 | LateWetPoor | | Late | | Wet | | Poor | | 706780 | | 6211031 | |  |
| 84 | MidWetPoor | | Mid | | Wet | | Poor | | 705403 | | 6209261 | |  |
| 85 | MidWetRich | | Mid | | Wet | | Rich | | 693213 | | 6212931 | |  |
| 86 | Spruce | | - | | - | | - | | 691428 | | 6215259 | |  |
| 87 | Oak | | - | | - | | - | | 702774 | | 6209432 | |  |
| 88 | EarlyDryRich | | Early | | Dry | | Rich | | 652603 | | 6189462 | |  |
| 89 | EarlyMoistPoor | | Early | | Moist | | Poor | | 643345 | | 6179060 | |  |
| 90 | EarlyWetPoor | | Early | | Wet | | Poor | | 642881 | | 6178095 | |  |
| 91 | EarlyMoistRich | | Early | | Moist | | Rich | | 638344 | | 6175468 | |  |
| 92 | EarlyWetRich | | Early | | Wet | | Rich | | 640495 | | 6175364 | |  |
| 93 | MidDryPoor | | Mid | | Dry | | Poor | | 651753 | | 6190536 | |  |
| 94 | MidDryRich | | Mid | | Dry | | Rich | | 618894 | | 6178182 | |  |
| 95 | Oldfield | | - | | - | | - | | 619112 | | 6178861 | |  |
| 96 | HighSpcRich | | - | | - | | - | | 675264 | | 6155726 | |  |
| 97 | LateDryRich | | Late | | Dry | | Rich | | 661990 | | 6140047 | |  |
| 98 | Beech | | - | | - | | - | | 664334 | | 6140553 | |  |
| 99 | LateMoistPoor | | Late | | Moist | | Poor | | 681900 | | 6161530 | |  |
| 100 | MidMoistPoor | | Mid | | Moist | | Poor | | 681902 | | 6160067 | |  |
| 101 | LateWetRich | | Late | | Wet | | Rich | | 661358 | | 6139741 | |  |
| 102 | Ley | | - | | - | | - | | 660682 | | 6140028 | |  |
| 103 | Rotational | | - | | - | | - | | 660837 | | 6132878 | |  |
| 104 | MidMoistRich | | Mid | | Moist | | Rich | | 664168 | | 6142266 | |  |
| 105 | LateDryPoor | | Late | | Dry | | Poor | | 579564 | | 6109181 | |  |
| 106 | EarlyDryRich | | Early | | Dry | | Rich | | 579489 | | 6109958 | |  |
| 107 | Oak | | - | | - | | - | | 579910 | | 6110352 | |  |
| 108 | EarlyWetPoor | | Early | | Wet | | Poor | | 583997 | | 6109030 | |  |
| 109 | MidDryPoor | | Mid | | Dry | | Poor | | 595693 | | 6107075 | |  |
| 110 | LateMoistPoor | | Late | | Moist | | Poor | | 587285 | | 6110023 | |  |
| 111 | MidMoistPoor | | Mid | | Moist | | Poor | | 587265 | | 6110673 | |  |
| 112 | MidWetPoor | | Mid | | Wet | | Poor | | 592504 | | 6112615 | |  |
| 113 | Spruce | | - | | - | | - | | 680392 | | 6073974 | |  |
| 114 | HighSpcRich | | - | | - | | - | | 680771 | | 6074624 | |  |
| 115 | Rotational | | - | | - | | - | | 660056 | | 6070320 | |  |
| 116 | LateWetPoor | | Late | | Wet | | Poor | | 663386 | | 6067126 | |  |
| 117 | LateWetRich | | Late | | Wet | | Rich | | 664394 | | 6070180 | |  |
| 118 | MidWetRich | | Mid | | Wet | | Rich | | 664221 | | 6069485 | |  |
| 119 | MidMoistRich | | Mid | | Moist | | Rich | | 670416 | | 6066245 | |  |
| 120 | Oldfield | | - | | - | | - | | 667308 | | 6069260 | |  |
| 121 | LateMoistRich | | Late | | Moist | | Rich | | 726446 | | 6098351 | |  |
| 122 | EarlyDryPoor | | Early | | Dry | | Poor | | 708859 | | 6105766 | |  |
| 123 | EarlyMoistPoor | | Early | | Moist | | Poor | | 708942 | | 6104123 | |  |
| 124 | EarlyMoistRich | | Early | | Moist | | Rich | | 720915 | | 6096629 | |  |
| 125 | EarlyWetRich | | Early | | Wet | | Rich | | 721199 | | 6095874 | |  |
| 126 | MidDryRich | | Mid | | Dry | | Rich | | 724661 | | 6096492 | |  |
| 127 | LateDryRich | | Late | | Dry | | Rich | | 726525 | | 6096762 | |  |
| 128 | HighSpcRich | | - | | - | | - | | 725620 | | 6098928 | |  |
| 129 | Ley | | - | | - | | - | | 710898 | | 6102472 | |  |
| 130 | Beech | | - | | - | | - | | 726483 | | 6097507 | |  |
