# Supplementary material for: A systematic survey of regional multi-taxon biodiversity: evaluating strategies and coverage
Source: BMC Ecol. 2019 Oct 15;19:43. doi: 10.1186/s12898-019-0260-x (PMC6792264; doi:10.1186/s12898-019-0260-x)
Supplement: Supplementary file 3 — Additional file 3: Appendix C: Ranges of environmental (abiotic and biotic) variables measured within the 130 sites as well as species richness of various taxonomic groups. [file 12898_2019_260_MOESM3_ESM.docx]

**Appendix C:** Ranges of environmental (abiotic and biotic) variables measured within the 130 sites as well as species richness of various taxonomic groups.

| Measured variable | Min value | Max value |
| --- | --- | --- |
| Litter mass from four litter samples from each site (21x21cm frame) (g/m^2^) | 0.57 | 2738.95 |
| Soil pH in 0-10 cm soil sample. Weighted for the proportion of organic matter to mineral soil | 3.94 | 8.13 |
| Soil C 0-10 cm (g/m^2^ ) | 6.26 | 21270.62 |
| Soil N 0-10 cm (g/m^2^ ) | 5.67 | 703.82 |
| Soil P 0-10 cm (g/m^2^) | 0.15 | 115.22 |
| Soil class (four classes: sand, sand-clay, organic, clay) | - | - |
| % leaf N - mean of four quadrants | 1.29 | 4.95 |
| % leaf C - mean of four quadrants | 40.55 | 47.98 |
| % leaf P - mean of four quadrants | 0.09 | 0.84 |
| Maximum surface temperature (°C) calculated for all day | 14.33 | 61.74 |
| Minimum nighttime air temperature (°C) | -3.30 | 8.42 |
| Median light intensity calculated for all day (lux) | 16.00 | 1344.00 |
| Mean daytime vapour pressure deficit (kPa) | 0.06 | 0.86 |
| Mean site Ellenberg L | 3.63 | 7.75 |
| Mean site Ellenberg F | 3.70 | 8.73 |
| Mean site Ellenberg R | 2.31 | 6.80 |
| Mean site Ellenberg N | 1.51 | 6.81 |
| Mean site Ellenberg S | 0.00 | 0.66 |
| Mean site Ellenberg T | 3.92 | 6.00 |
| Trimmed mean soil moisture (%Volumetric Water Content (VWC)) value from 16 soil moisture meter values pr. site | 1.28 | 77.72 |
| Species number of plants in site | 11.00 | 134.00 |
| Species number of bryophytes in site | 0.00 | 50.00 |
| Species number of macrofungi in site | 0.00 | 180.00 |
| Species number of lichen in site | 0.00 | 33.00 |
| Species number of carabid beetles in site | 0.00 | 21.00 |
| Species number of hoverflies in site | 0.00 | 22.00 |
| Species number of spiders in site | 7.00 | 53.00 |
| Species number of gallers and miners in site | 0.00 | 17.00 |
| Species number of gastropods in site | 0.00 | 33.00 |
| Mean herb layer vegetation height (cm, four measurements within each of the four plots) | 0.00 | 146.69 |
| Mean % bare soil (mean of % cover in each of the four 5 m-circle plots) | 0.00 | 97.00 |
| Mean % bryophyte cover (mean of % cover in each of the four 5 m-circle plots) | 0.00 | 95.00 |
| Mean % lichen cover (mean of % cover in each of the four 5 m-circle plots) | 0.00 | 54.25 |
| Volume of dead wood (m^3^/ha) | 0.00 | 8.14 |
| Estimated density of trees < 40DBH – deciduous (number/m^2^) | 0.00 | 0.49 |
| Estimated density of trees < 40DBH - coniferous (number/m^2^) | 0.00 | 0.08 |
| Number of trees > 40DBH | 0.00 | 24.00 |
| Number of uprooted trees | 0.00 | 13.00 |
| Number of carcasses | 0.00 | 0.00 |
| Basic distance abundance estimate of deer dung (number/m^2^). BADV3 from White et al. (2008) | 0.00 | 0.98 |
| Basic distance abundance estimate of hare dung (number/m^2^). BADV3 from White et al. (2008) | 0.00 | 7.07 |
| Basic distance abundance estimate of cow dung (number/m^2^). BADV3 from White et al. (2008) | 0.00 | 0.24 |
| Basic distance abundance estimate of sheep dung (number/m^2^). BADV3 from White et al. (2008) | 0.00 | 3.89 |
| Basic distance abundance estimate of horse dung (number/m^2^). BADV3 from White et al. (2008) | 0.00 | 0.27 |
| Summed dung density (number/m^2^). BADV3 from White et al. (2008) | 0.00 | 7.07 |
| Basic distance abundance estimate of water puddles > 0.25m^2^ (number/m^2^). BADV3 from White et al. (2008) | 0.00 | 0.19 |
| Basic distance abundance estimate of dead wood (diameter 5-20 cm and length >1m or diameter >20cm and length <1m) (number/m^2^). BADV3 from White et al. (2008) | 0.00 | 3.02 |
| Basic distance abundance estimate of ant hills (height > 10cm) (number/m^2^). BADV3 from White et al. (2008) | 0.00 | 0.08 |
| Basic distance abundance estimate of boulders (diameter > 20cm) (number/m^2^). BADV3 from White et al. (2008) | 0.00 | 0.16 |
| Estimated mean flower abundance within site (number/m^2^). BADV3 from White et al. (2008) | 0.00 | 1223 |
| Temporal continuity class (4 levels: 1: <15 years. 2: 15-45 years. 3: 45-135 years. 4: >135 years) | 1.00 | 4.00 |
| Spatial continuity of focal habitat type within 500 m buffer (%) | 2.00 | 93.33 |
| Spatial continuity of focal habitat type within 1000 m buffer (%) | 1.00 | 78.33 |
| Spatial continuity of focal habitat type within 2000 m buffer (%) | 0.03 | 73.33 |
| Spatial continuity of focal habitat type within 5000 m buffer (%) | 0.03 | 71.67 |
