# Supplementary material for: A systematic survey of regional multi-taxon biodiversity: evaluating strategies and coverage
Source: BMC Ecol. 2019 Oct 15;19:43. doi: 10.1186/s12898-019-0260-x (PMC6792264; doi:10.1186/s12898-019-0260-x)
Supplement: Supplementary file 4 — Additional file 4: Appendix D. Temporal and spatial continuity for the 130 Biowide sites. [file 12898_2019_260_MOESM4_ESM.docx]

**Appendix D:** Temporal and spatial continuity for the 130 Biowide sites. Temporal continuity is represented on a 4-level ordinal scale: 1: < 15 years of continuity, 2: 15-44 years of continuity, 3: 45-135 years of continuity and 4: >135 years of continuity (or continous on the oldest available map). Spatial continuity represents the amount of similar habitat (%) as the focal site habitat within four different buffer sizes (500m, 1000m, 2000m, 5000m).

**
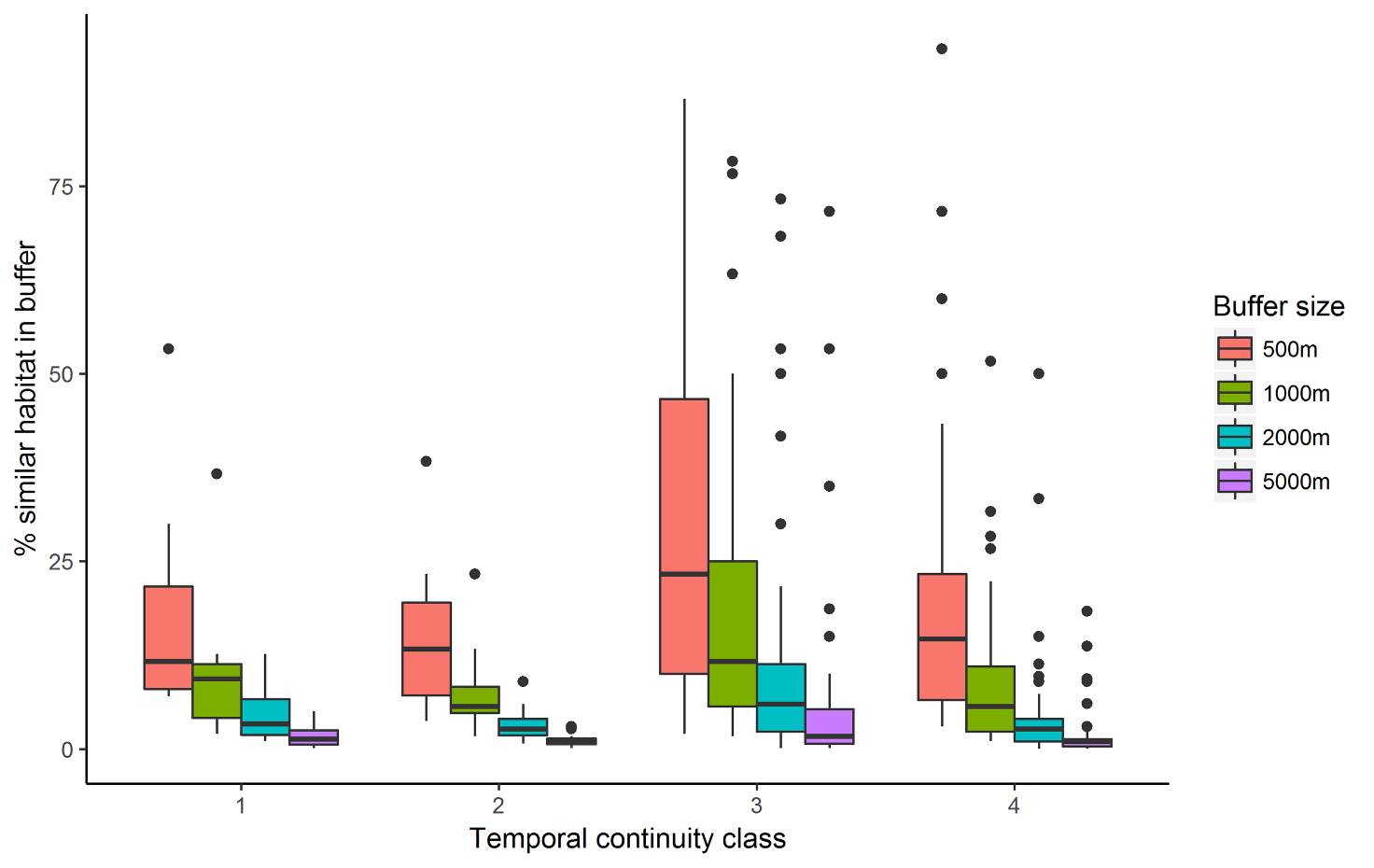
**
