# Supplementary material for: A systematic survey of regional multi-taxon biodiversity: evaluating strategies and coverage
Source: BMC Ecol. 2019 Oct 15;19:43. doi: 10.1186/s12898-019-0260-x (PMC6792264; doi:10.1186/s12898-019-0260-x)
Supplement: Supplementary file 5 — Additional file 5: Appendix E. Relative richness of arthropods, bryophytes, gastropods, lichens, macrofungi and vascular plants across all 130 sites. [file 12898_2019_260_MOESM5_ESM.docx]

**Appendix E:** Relative richness of arthropods, bryophytes, gastropods, lichens, macrofungi and vascular plants across the five geographical regions: Eastern Jutland (Ejut), Funen, Lolland, Møn (FLM), Northern Jutland (Njut), Western Jutland (Wjut) and Zealand (Zeal). Nestedness and turnover for each region as well as average and standard deviations are given.

**
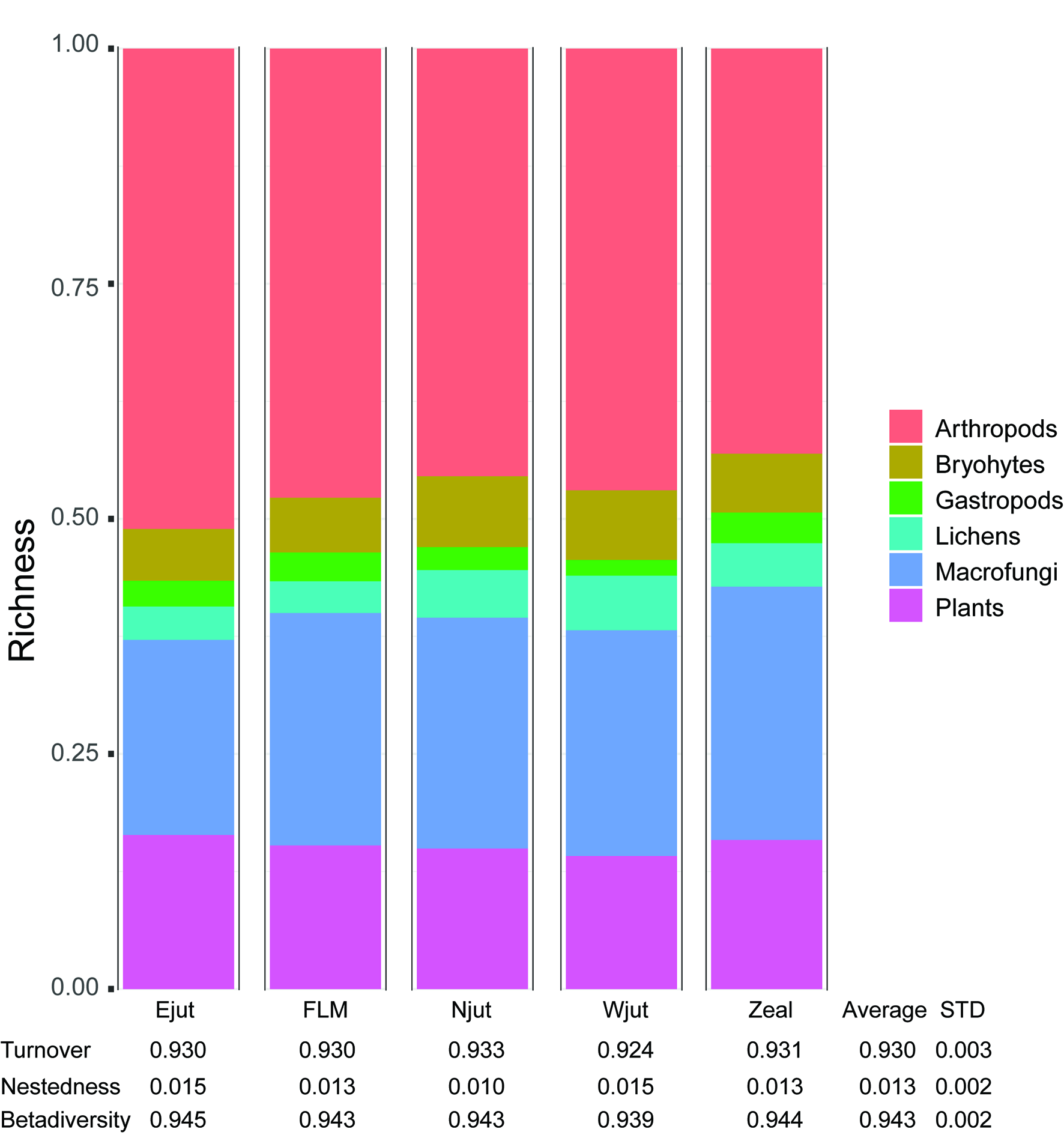
**
