# Supplementary material for: A systematic survey of regional multi-taxon biodiversity: evaluating strategies and coverage
Source: BMC Ecol. 2019 Oct 15;19:43. doi: 10.1186/s12898-019-0260-x (PMC6792264; doi:10.1186/s12898-019-0260-x)
Supplement: Supplementary file 6 — Additional file 6: Appendix F. Number of species in each arthropod family for natural habitats, perceived areas of high species richness, arable land and plantations. [file 12898_2019_260_MOESM6_ESM.docx]

**Appendix F:** Number of species per arthropod family found in natural sites (n=90), perceived areas of high species richness (HighSpcRich, n=10), arable land (n=15), and plantations (n=15). The number of unique species for each habitat type and family is given in brackets.

| Order | Family | Total | Natural | HighSpcRich | Arable | Plantation |
| --- | --- | --- | --- | --- | --- | --- |
| Araneae | Agelenidae | 2 | 2 (0) | 1 (0) | 1 (0) | 1 (0) |
| Araneae | Amaurobiidae | 2 | 2 (0) |  |  | 2 (0) |
| Araneae | Anyphaenidae | 1 | 1 (0) | 1 (0) |  | 1 (0) |
| Araneae | Araneidae | 23 | 21 (5) | 13 (0) | 10 (0) | 8 (2) |
| Araneae | Atypidae | 1 | 1 (0) | 1 (0) |  |  |
| Araneae | Clubionidae | 13 | 13 (3) | 8 (0) | 7 (0) | 4 (0) |
| Araneae | Corinnidae | 2 | 1 (0) | 2 (1) |  |  |
| Araneae | Cybaeidae | 1 | 1 (1) |  |  |  |
| Araneae | Dictynidae | 4 | 4 (1) | 2 (0) | 2 (0) | 1 (0) |
| Araneae | Gphosidae | 17 | 17 (6) | 10 (0) | 4 (0) | 1 (0) |
| Araneae | Hahniidae | 4 | 4 (1) | 3 (0) |  | 2 (0) |
| Araneae | Linyphiidae | 144 | 132 (44) | 50 (1) | 54 (2) | 72 (8) |
| Araneae | Liocranidae | 4 | 4 (3) | 1 (0) |  | 1 (0) |
| Araneae | Lycosidae | 25 | 25 (4) | 17 (1) | 14 (0) | 3 (0) |
| Araneae | Mimetidae | 2 | 2 (1) |  | 1 (0) | 1 (0) |
| Araneae | Miturgidae | 2 | 2 (0) | 2 (0) | 2 (0) |  |
| Araneae | Oxyopidae | 1 |  | 1 (1) |  |  |
| Araneae | Philodromidae | 9 | 8 (2) | 5 (0) | 4 (0) | 4 (0) |
| Araneae | Pisauridae | 2 | 3 (1) | 1 (0) | 1 (0) |  |
| Araneae | Salticidae | 18 | 16 (11) | 3 (0) | 4 (0) | 2 (1) |
| Araneae | Segestriidae | 1 | 1 (0) |  |  | 1 (0) |
| Araneae | Sparassidae | 1 | 1 (1) |  |  |  |
| Araneae | Tetragthidae | 11 | 11 (1) | 9 (0) | 3 (0) | 8 (0) |
| Araneae | Theridiidae | 30 | 27 (11) | 12 (0) | 15 (2) | 10 (0) |
| Araneae | Theridiosomatidae | 1 | 1 (1) |  |  |  |
| Araneae | Thomisidae | 12 | 11 (5) | 5 (1) | 3 (0) | 3 (1) |
| Araneae | Uloboridae | 1 | 1 (0) |  |  | 1 (0) |
| Araneae | Zoridae | 1 | 1 (0) |  | 1 (0) | 1 (0) |
| Coleoptera | Anthribidae | 2 | 2 (1) | 1 (0) |  | 1 (0) |
| Coleoptera | Attelabidae | 7 | 5 (5) |  |  | 2 (2) |
| Coleoptera | Brentidae | 34 | 30 (13) | 8 (1) | 19 (3) | 1 (0) |
| Coleoptera | Buprestidae | 1 | 1 (1) |  |  |  |
| Coleoptera | Cantharidae | 34 | 32 (11) | 10 (0) | 10 (0) | 11 (2) |
| Coleoptera | Carabidae | 123 | 107 (43) | 34 (3) | 51 (15) | 35 (1) |
| Coleoptera | Cerambycidae | 12 | 12 (6) | 2 (0) | 1 (0) | 5 (1) |
| Coleoptera | Cleridae | 2 | 2 (2) |  |  |  |
| Coleoptera | Coccinellidae | 20 | 20 (9) | 5 (0) | 8 (0) | 5 (0) |
| Coleoptera | Curculionidae | 132 | 104 (55) | 35 (9) | 43 (13) | 32 (6) |
| Coleoptera | Dascillidae | 1 |  | 1 (1) |  |  |
| Coleoptera | Dasytidae | 2 | 2 (2) |  |  |  |
| Coleoptera | Drilidae | 1 | 1 (1) |  |  |  |
| Coleoptera | Elateridae | 22 | 21 (6) | 9 (0) | 7 (0) | 6 (1) |
| Coleoptera | Endomychidae | 1 |  |  | 1 (1) |  |
| Coleoptera | Eucnemidae | 2 | 2 (1) | 1 (0) |  | 1 (0) |
| Coleoptera | Geotrupidae | 3 | 3 (1) | 2 (0) | 2 (0) | 2 (0) |
| Coleoptera | Histeridae | 5 | 4 (2) | 2 (1) | 2 (0) |  |
| Coleoptera | Hydrophilidae | 11 | 10 (3) | 6 (1) | 5 (0) |  |
| Coleoptera | Kateretidae | 1 | 1 (1) |  |  |  |
| Coleoptera | Lampyridae | 1 | 1 (1) |  |  |  |
| Coleoptera | Latridiidae | 2 | 2 (1) |  |  | 1 (0) |
| Coleoptera | Lucanidae | 1 | 1 (1) |  |  |  |
| Coleoptera | Lycidae | 2 | 2 (0) |  |  | 2 (0) |
| Coleoptera | Malachiidae | 2 | 2 (1) |  | 1 (0) |  |
| Coleoptera | Melandryidae | 1 | 1 (1) |  |  |  |
| Coleoptera | Monotomidae | 1 | 1 (1) |  |  |  |
| Coleoptera | Nitidulidae | 2 | 2 (1) |  | 1 (0) |  |
| Coleoptera | Oedemeridae | 3 | 3 (1) | 1 (0) |  | 1 (0) |
| Coleoptera | Ptinidae | 2 | 2 (2) |  |  |  |
| Coleoptera | Pyrochroidae | 1 | 1 (1) |  |  |  |
| Coleoptera | Salpingidae | 2 | 2 (1) |  |  | 1 (0) |
| Coleoptera | Scarabaeidae | 28 | 25 (8) | 13 (1) | 17 (2) | 6 (0) |
| Coleoptera | Silphidae | 10 | 10 (0) | 7 (0) | 8 (0) | 6 (0) |
| Coleoptera | Staphylinidae | 76 | 54 (29) | 15 (6) | 25 (15) | 16 (4) |
| Coleoptera | Tenebrionidae | 3 | 3 (2) | 1 (0) | 1 (0) |  |
| Coleoptera | Throscidae | 1 | 2 (1) |  |  |  |
| Diptera | Acroceridae | 1 | 1 (0) |  | 1 (0) |  |
| Diptera | Asilidae | 8 | 7 (2) | 4 (0) | 2 (1) | 2 (0) |
| Diptera | Bombyliidae | 2 | 1 (1) | 1 (1) |  |  |
| Diptera | Cecidomyiidae | 1 | 1 (1) |  |  |  |
| Diptera | Chloropidae | 2 | 2 (2) |  |  |  |
| Diptera | Hybotidae | 1 | 1 (1) |  |  |  |
| Diptera | Micropezidae | 1 |  |  | 1 (1) |  |
| Diptera | Oestridae | 1 | 1 (1) |  |  |  |
| Diptera | Pediciidae | 1 | 1 (1) |  |  |  |
| Diptera | Platystomatidae | 2 | 1 (0) | 2 (1) | 1 (0) |  |
| Diptera | Ptychopteridae | 1 | 1 (1) |  |  |  |
| Diptera | Rhagionidae | 5 | 5 (0) | 3 (0) | 3 (0) | 5 (0) |
| Diptera | Rhinophoridae | 4 | 4 (3) | 1 (0) |  |  |
| Diptera | Scathophagidae | 1 | 1 (1) |  |  |  |
| Diptera | Sciomyzidae | 2 | 2 (2) |  |  |  |
| Diptera | Stratiomyidae | 14 | 14 (8) | 4 (1) | 6 (0) |  |
| Diptera | Syrphidae | 98 | 93 (41) | 36 (2) | 45 (6) | 20 (2) |
| Diptera | Tachinidae | 28 | 26 (14) | 7 (2) | 8 (1) | 8 (2) |
| Diptera | Tephritidae | 20 | 15 (7) | 5 (2) | 9 (3) |  |
| Diptera | Ulidiidae | 2 | 2 (2) |  |  |  |
| Diptera | Xylomyidae | 1 | 1 (1) |  |  |  |
| Hemiptera | Acanthosomatidae | 5 | 6 (3) | 1 (1) | 1 (0) | 2 (0) |
| Hemiptera | Alydidae | 1 | 1 (1) |  |  |  |
| Hemiptera | Anthocoridae | 10 | 11 (3) | 7 (1) | 5 (0) | 3 (0) |
| Hemiptera | Aphrophoridae | 5 | 5 (1) | 4 (0) | 2 (0) | 1 (0) |
| Hemiptera | Berytidae | 3 | 3 (0) | 2 (0) | 1 (0) | 1 (0) |
| Hemiptera | Caliscelidae | 1 | 1 (1) |  |  |  |
| Hemiptera | Ceratocombidae | 1 | 1 (0) | 1 (0) |  | 1 (0) |
| Hemiptera | Cercopidae | 1 | 1 (1) |  |  |  |
| Hemiptera | Cicadellidae | 158 | 169 (52) | 80 (3) | 85 (6) | 44 (2) |
| Hemiptera | Cimicidae | 1 | 1 (1) |  |  |  |
| Hemiptera | Cixiidae | 4 | 5 (2) |  | 2 (0) | 3 (0) |
| Hemiptera | Coreidae | 1 | 1 (1) |  |  |  |
| Hemiptera | Cydnidae | 3 | 3 (3) |  |  |  |
| Hemiptera | Delphacidae | 32 | 38 (20) | 11 (1) | 8 (1) | 4 (0) |
| Hemiptera | Gerridae | 1 | 1 (1) |  |  |  |
| Hemiptera | Lygaeidae | 33 | 35 (17) | 13 (2) | 5 (0) | 6 (1) |
| Hemiptera | Microphysidae | 4 | 5 (1) | 4 (0) | 2 (0) | 3 (0) |
| Hemiptera | Miridae | 113 | 112 (47) | 40 (4) | 34 (1) | 31 (4) |
| Hemiptera | bidae | 10 | 10 (1) | 9 (0) | 6 (1) | 5 (0) |
| Hemiptera | Nepidae | 1 | 1 (1) |  |  |  |
| Hemiptera | Pemphigidae | 1 | 1 (1) |  |  |  |
| Hemiptera | Pentatomidae | 15 | 16 (8) | 6 (0) | 4 (0) | 1 (0) |
| Hemiptera | Piesmatidae | 1 | 1 (0) |  | 1 (0) |  |
| Hemiptera | Psyllidae | 5 | 4 (2) | 2 (1) | 1 (0) |  |
| Hemiptera | Reduviidae | 4 | 3 (3) |  |  | 1 (1) |
| Hemiptera | Rhopalidae | 6 | 7 (2) | 3 (0) | 3 (0) |  |
| Hemiptera | Saldidae | 10 | 11 (9) |  | 2 (0) | 1 (0) |
| Hemiptera | Scutelleridae | 2 | 3 (2) |  |  |  |
| Hemiptera | Tingidae | 10 | 10 (2) | 6 (0) | 5 (0) |  |
| Hemiptera | Triozidae | 1 | 1 (0) | 1 (0) | 1 (0) |  |
| Hemiptera | Veliidae | 2 | 2 (2) |  |  |  |
| Hymenoptera | Ampulicidae | 1 |  | 1 (1) |  |  |
| Hymenoptera | Andrenidae | 5 | 2 (0) | 2 (1) | 2 (0) | 3 (1) |
| Hymenoptera | Aphelinidae | 1 | 1 (1) |  |  |  |
| Hymenoptera | Apidae | 14 | 13 (6) | 7 (1) | 5 (0) | 6 (0) |
| Hymenoptera | Argidae | 2 | 3 (2) |  |  |  |
| Hymenoptera | Bethylidae | 1 |  |  | 1 (1) |  |
| Hymenoptera | Chalcididae | 2 | 1 (1) | 1 (1) |  |  |
| Hymenoptera | Chrysididae | 2 | 2 (2) |  |  |  |
| Hymenoptera | Colletidae | 4 | 4 (1) | 2 (0) | 2 (0) |  |
| Hymenoptera | Crabronidae | 29 | 31 (18) | 11 (1) | 5 (1) | 3 (0) |
| Hymenoptera | Cynipidae | 3 | 3 (3) |  |  |  |
| Hymenoptera | Diapriidae | 1 |  |  |  | 1 (1) |
| Hymenoptera | Dryinidae | 2 | 2 (0) |  | 1 (0) | 2 (0) |
| Hymenoptera | Encyrtidae | 1 | 1 (1) |  |  |  |
| Hymenoptera | Evaniidae | 1 | 1 (0) | 1 (0) |  |  |
| Hymenoptera | Formicidae | 1 | 2 (1) |  |  |  |
| Hymenoptera | Halictidae | 12 | 11 (7) | 4 (2) | 2 (0) |  |
| Hymenoptera | Ichneumonidae | 3 | 3 (2) | 1 (0) |  |  |
| Hymenoptera | Megachilidae | 9 | 6 (5) | 3 (2) | 1 (1) |  |
| Hymenoptera | Megaspilidae | 1 | 2 (1) |  |  |  |
| Hymenoptera | Melittidae | 2 | 1 (1) |  | 1 (1) |  |
| Hymenoptera | Mutillidae | 1 | 1 (1) |  |  |  |
| Hymenoptera | Mymaridae | 1 | 1 (1) |  |  |  |
| Hymenoptera | Pamphiliidae | 1 |  |  |  | 1 (1) |
| Hymenoptera | Pompilidae | 11 | 12 (8) | 2 (1) | 2 (1) |  |
| Hymenoptera | Pteromalidae | 1 | 1 (1) |  |  |  |
| Hymenoptera | Sphecidae | 4 | 4 (3) | 1 (0) |  |  |
| Hymenoptera | Tenthredinidae | 56 | 54 (31) | 11 (4) | 14 (1) | 8 (2) |
| Hymenoptera | Tiphiidae | 1 | 1 (1) |  |  |  |
| Hymenoptera | Vespidae | 13 | 14 (6) | 5 (0) | 3 (0) | 4 (0) |
| Lepidoptera | Drepanidae | 2 | 2 (1) | 1 (0) |  |  |
| Lepidoptera | Erebidae | 4 | 4 (2) | 1 (0) |  | 1 (0) |
| Lepidoptera | Geometridae | 16 | 12 (6) | 5 (2) |  | 5 (2) |
| Lepidoptera | Hepialidae | 3 | 3 (2) | 1 (0) | 1 (0) |  |
| Lepidoptera | Hesperiidae | 3 | 3 (3) |  |  |  |
| Lepidoptera | Lasiocampidae | 1 | 1 (1) |  |  |  |
| Lepidoptera | Limacodidae | 1 | 1 (1) |  |  |  |
| Lepidoptera | Lycaenidae | 3 | 3 (2) |  | 1 (0) |  |
| Lepidoptera | Noctuidae | 74 | 75 (39) | 20 (1) | 25 (2) | 10 (0) |
| Lepidoptera | Nymphalidae | 10 | 10 (6) | 2 (0) | 4 (0) |  |
| Lepidoptera | Pieridae | 3 | 3 (3) |  |  |  |
| Lepidoptera | Pyralidae | 1 | 1 (1) |  |  |  |
| Lepidoptera | Sphingidae | 1 | 1 (1) |  |  |  |
| Lepidoptera | Tortricidae | 1 | 2 (1) |  |  |  |
| Lepidoptera | Zygaenidae | 4 | 3 (2) | 1 (0) | 1 (1) |  |
| Neuroptera | Chrysopidae | 10 | 7 (3) | 1 (0) | 4 (0) | 5 (3) |
| Neuroptera | Coniopterygidae | 3 | 3 (0) | 3 (1) | 1 (0) |  |
| Neuroptera | Hemerobiidae | 9 | 10 (4) | 5 (0) | 2 (0) | 2 (0) |
| Neuroptera | Sisyridae | 1 | 1 (1) |  |  |  |
| Opiliones | Nemastomatidae | 1 | 1 (0) | 1 (0) | 1 (0) | 1 (0) |
| Opiliones | Phalangiidae | 16 | 15 (2) | 10 (0) | 8 (0) | 13 (0) |
| Opiliones | Trogulidae | 1 | 1 (1) |  |  |  |
| Orthoptera | Acrididae | 9 | 9 (2) | 5 (0) | 7 (1) |  |
| Orthoptera | Phaneropteridae | 1 | 1 (0) |  | 1 (0) |  |
| Orthoptera | Tetrigidae | 2 | 2 (2) |  |  |  |
| Orthoptera | Tettigoniidae | 8 | 8 (1) | 6 (0) | 2 (0) | 3 (0) |
| Plecoptera | Taeniopterygidae | 1 | 1 (1) |  |  |  |
| Prostigmata | Eriophyidae | 4 | 4 (4) |  |  |  |
| Prostigmata | Erythraeidae | 1 |  | 2 (1) |  |  |
| Psocoptera | Amphipsocidae | 1 | 1 (0) | 1 (0) | 1 (0) |  |
| Psocoptera | Caeciliusidae | 8 | 9 (1) | 5 (0) | 4 (0) | 4 (0) |
| Psocoptera | Elipsocidae | 5 | 6 (2) | 4 (0) | 4 (0) | 2 (0) |
| Psocoptera | Lachesillidae | 1 | 1 (0) | 1 (0) | 1 (0) | 1 (0) |
| Psocoptera | Mesopsocidae | 3 | 3 (1) | 1 (0) | 2 (0) |  |
| Psocoptera | Peripsocidae | 5 | 6 (1) | 2 (0) | 4 (0) | 2 (0) |
| Psocoptera | Philotarsidae | 2 | 2 (0) | 1 (0) | 1 (0) | 2 (0) |
| Psocoptera | Psocidae | 8 | 7 (1) | 4 (0) | 6 (0) | 4 (1) |
| Psocoptera | Stenopsocidae | 2 | 3 (1) | 2 (0) | 2 (0) | 2 (0) |
| Psocoptera | Trogiidae | 2 | 2 (1) | 1 (0) | 1 (0) | 1 (0) |
| Raphidioptera | Raphidiidae | 2 | 2 (2) |  |  |  |
| Strepsiptera | Elenchidae | 1 | 1 (0) |  | 1 (0) | 1 (0) |
| Strepsiptera | Halictophagidae | 1 | 1 (1) |  |  |  |
| Trichoptera | Beraeidae | 3 | 2 (1) | 1 (1) |  | 1 (0) |
| Trichoptera | Ecnomidae | 1 | 1 (0) |  | 1 (0) |  |
| Trichoptera | Hydropsychidae | 2 | 1 (1) |  | 1 (1) |  |
| Trichoptera | Hydroptilidae | 7 | 7 (4) |  | 3 (0) |  |
| Trichoptera | Lepidostomatidae | 1 | 1 (1) |  |  |  |
| Trichoptera | Leptoceridae | 12 | 11 (8) | 2 (0) | 3 (1) |  |
| Trichoptera | Limnephilidae | 36 | 36 (15) | 15 (0) | 13 (0) | 11 (1) |
| Trichoptera | Molannidae | 2 | 2 (2) |  |  |  |
| Trichoptera | Phryganeidae | 6 | 6 (1) | 3 (0) | 2 (0) | 1 (0) |
| Trichoptera | Polycentropodidae | 6 | 6 (3) | 2 (0) | 1 (0) | 2 (0) |
| Trichoptera | Psychomyiidae | 3 | 3 (3) |  |  |  |
| Trichoptera | Sericostomatidae | 1 | 1 (0) |  |  | 1 (0) |
