# Supplementary figures and images for: A systematic survey of regional multi-taxon biodiversity: evaluating strategies and coverage
Source: BMC Ecol. 2019 Oct 15;19:43. doi: 10.1186/s12898-019-0260-x (PMC6792264; doi:10.1186/s12898-019-0260-x)

**Appendix G:** Species accumulation curves for arable sites (n=15), plantations (n=15) and natural sites (n=90).

**
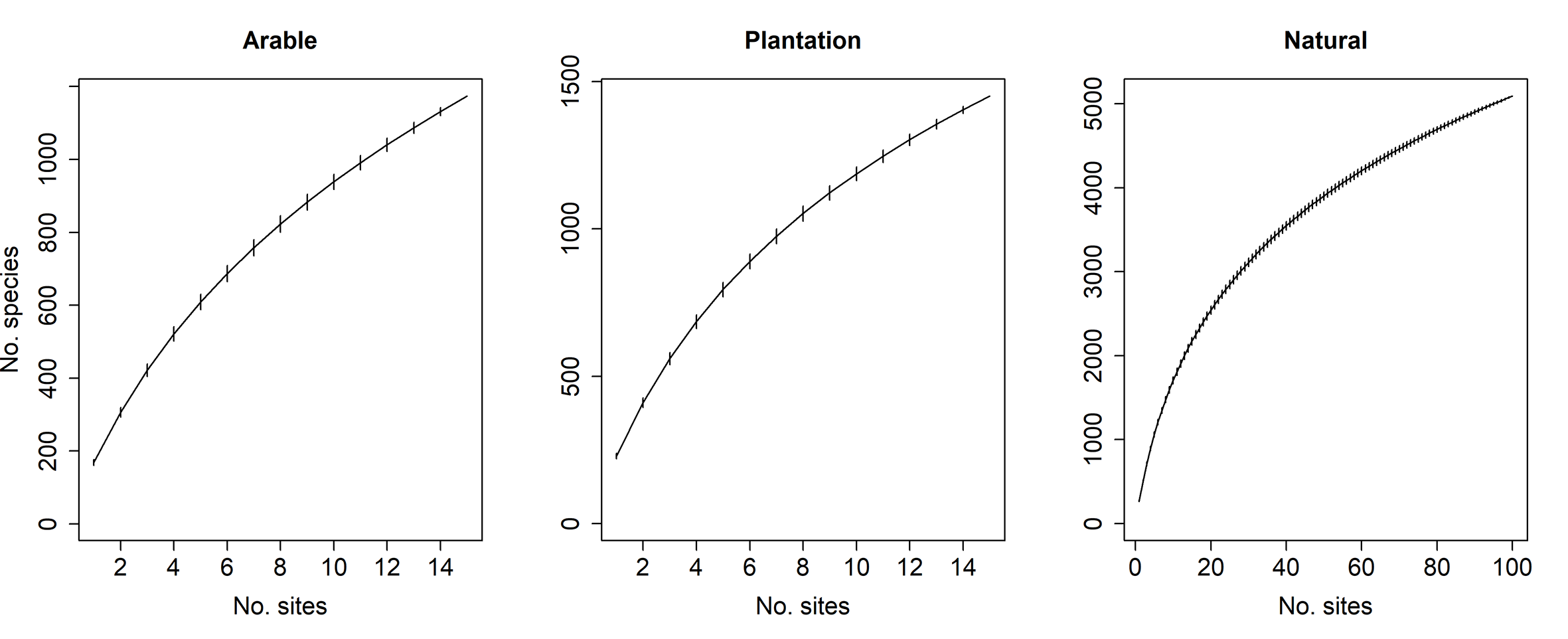
**

Supplement: Supplementary file 7 — Additional file 7: Appendix G. Species accumulation curves for arable sites, plantations and natural sites. [file 12898_2019_260_MOESM7_ESM.docx]
