# Supplementary material for: A systematic survey of regional multi-taxon biodiversity: evaluating strategies and coverage
Source: BMC Ecol. 2019 Oct 15;19:43. doi: 10.1186/s12898-019-0260-x (PMC6792264; doi:10.1186/s12898-019-0260-x)
Supplement: Supplementary file 8 — Additional file 8: Appendix H. Correlation matrix for NMDS axes 1, 2 and 3 and environmental variables. [file 12898_2019_260_MOESM8_ESM.docx]

**Appendix H:** Spearman correlation (r) between measured abiotic and biotic environmental variables and Nonmetric Multidimensional Scaling (NMDS) plant and macrofungi axes 1-3. N = 130 for plant NMDS and N = 124 for macrofungi NMDS (sites with less than five species were omitted). Bold indicates significance at 0.05 level.

|  | PlantNMS1 | PlantNMS2 | PlantNMS3 | FungiNMS1 | FungiNMS2 | FungiNMS3 |
| --- | --- | --- | --- | --- | --- | --- |
| Litter mass | **-0.302** | **-0.730** | 0.040 | **0.652** | **0.341** | 0.106 |
| Soil pH | **0.661** | 0.079 | 0.026 | **-0.432** | **0.475** | 0.076 |
| Soil C | 0.058 | **0.189** | 0.028 | **-0.203** | -0.030 | 0.029 |
| Soil N | **0.580** | -0.002 | **0.255** | **-0.363** | **0.274** | **0.296** |
| Soil P | **0.733** | -0.103 | **0.205** | **-0.428** | **0.349** | **0.317** |
| Leaf N | **0.361** | **-0.529** | **-0.266** | 0.054 | **0.542** | -0.065 |
| Leaf C | **-0.507** | **0.368** | -0.066 | 0.063 | **-0.527** | **-0.198** |
| Leaf P | **0.509** | **-0.219** | -0.023 | **-0.246** | **0.376** | 0.175 |
| Deadwood volume | -0.141 | **-0.440** | 0.123 | **0.308** | **0.239** | 0.085 |
| Mean vegetation height | **0.240** | 0.036 | **-0.425** | -0.156 | **0.161** | **-0.321** |
| Trimmed mean soil moisture | **-0.211** | 0.121 | **-0.842** | -0.035 | **0.029** | **-0.703** |
| Max surface temperature | 0.063 | **0.583** | **0.228** | **-0.417** | **-0.376** | 0.147 |
| Sd air temperature | 0.105 | **0.495** | 0.139 | **-0.516** | **-0.267** | 0.102 |
| 85 quantile Vapour Pressure Deficit | 0.087 | **0.441** | **0.326** | **-0.392** | **-0.303** | **0.238** |
| Median light intensity | 0.026 | **0.723** | 0.071 | **-0.462** | **-0.493** | -0.171 |
| Plant species richness | **0.483** | **0.209** | -0.036 | **-0.430** | **0.358** | 0.032 |
| Trees > 40DBH (count) | -0.095 | **-0.672** | 0.059 | **0.479** | **0.343** | 0.092 |
| Trees > 40DBH (no. of species) | -0.149 | **-0.685** | 0.091 | **0.500** | **0.300** | 0.134 |
| Mean bare soil cover | -0.168 | **-0.695** | 0.139 | **0.617** | **0.336** | 0.088 |
| Mean bryophyte cover | **-0.386** | **0.303** | -0.039 | 0.034 | **-0.523** | -0.053 |
| Mean lichen cover | -0.026 | **0.206** | **0.313** | -0.109 | **-0.231** | 0.119 |
| Deadwood density | -0.034 | -0.147 | -0.146 | 0.099 | 0.133 | -0.092 |
| Anthill density | -0.076 | 0.124 | 0.073 | -0.018 | -0.128 | 0.063 |
| Boulder density | 0.082 | -0.075 | 0.170 | 0.023 | 0.065 | 0.129 |
| Water puddle density | 0.035 | -0.011 | **-0.206** | -0.010 | 0.095 | **-0.191** |
| Dung density | 0.056 | **0.269** | **0.234** | **-0.219** | -0.146 | **0.266** |
| Flower density | 0.082 | 0.149 | 0.155 | -0.122 | -0.016 | 0.173 |
| Temporal continuity class | **-0.209** | 0.007 | 0.095 | 0.100 | -0.103 | 0.010 |
| Spatial continuity 500m | **0.275** | -0.117 | **0.291** | 0.094 | -0.039 | **0.378** |
| Spatial continuity 1000m | **0.355** | -0.102 | **0.182** | 0.070 | -0.095 | **0.368** |
| Spatial continuity 2000m | **0.377** | -0.085 | 0.125 | 0.069 | -0.139 | **0.342** |
| Spatial continuity 5000m | **0.468** | -0.082 | 0.054 | -0.038 | -0.115 | **0.358** |

**References**

1. Pansu M, Gautheyrou J: **Handbook of soil analysis: mineralogical, organic and inorganic methods**: Springer Science & Business Media; 2007.

2. Stenberg B, Viscarra Rossel RA, Mouazen AM, Wetterlind J: **Visible and near infrared spectroscopy in soil science**. In: *Advances in Agronomy.* Edited by Donald LS, vol. Volume 107: Academic Press; 2010: 163-215.

3. Jones HG: **Plants and microclimate: A quantitative approach to environmental plant physiology**. New York: Cambridge University Press; 1992.

4. **Leaf Transpiration Calculator. landflux.org. Available at: <http://landflux.org/resources/Ecofiz_E_K2_v5.xls>**

5. White N, Engeman R, Sugihara R, Krupa H: **A comparison of plotless density estimators using Monte Carlo simulation on totally enumerated field data sets**. *BMC Ecology* 2008, **8**(1):6.

6. Frøslev TG, Kjoller R, Bruun HH, Ejrnæs R, Brunbjerg AK, Pietroni C, Hansen AJ: **Algorithm for post-clustering curation of DNA amplicon data yields reliable biodiversity estimates**. *Nature Comm* 2017, **8**:1188.

7. Sagova-Mareckova M, Cermak L, Novotna J, Plhackova K, Forstova J, Kopecky J: **Innovative methods for soil DNA purification tested in soils with widely differing characteristics**. *Applied and Environmental Microbiology* 2008, **74**(9):2902-2907.

8. Chen S, Yao H, Han J, Liu C, Song J, Shi L, Zhu Y, Ma X, Gao T, Pang X *et al*: **Validation of the ITS2 Region as a Novel DNA Barcode for Identifying Medicinal Plant Species**. *PLoS One* 2010, **5**(1):e8613.

9. White TJ, Bruns T, Lee S, Taylor J: **Amplification and direct sequencing of fungal ribosomal RNA genes for phylogenetics**. *PCR protocols: a guide to methods and applications* 1990, **18**(1):315-322.

10. Taberlet P, Coissac E, Pompanon F, Gielly L, Miquel C, Valentini A, Vermat T, Corthier G, Brochmann C, Willerslev E: **Power and limitations of the chloroplast trn L (UAA) intron for plant DNA barcoding**. *Nucleic Acids Research* 2007, **35**(3):e14-e14.

11. Ihrmark K, Bödeker ITM, Cruz-Martinez K, Friberg H, Kubartova A, Schenck J, Strid Y, Stenlid J, Brandström-Durling M, Clemmensen KE *et al*: **New primers to amplify the fungal ITS2 region – evaluation by 454-sequencing of artificial and natural communities**. *FEMS Microbiology Ecology* 2012, **82**(3):666-677.

12. Bienert F, De Danieli S, Miquel C, Coissac E, Poillot C, Brun JJ, Taberlet P: **Tracking earthworm communities from soil DNA**. *Mol Ecol* 2012, **21**(8):2017-2030.

13. Guardiola M, Uriz MJ, Taberlet P, Coissac E, Wangensteen OS, Turon X: **Deep-sea, deep-sequencing: Metabarcoding extracellular DNA from sediments of marine canyons**. *PLoS One* 2015, **10**(10):e0139633.

14. Simon L, Lalonde M, Bruns TD: **Specific amplification of 18S fungal ribosomal genes from vesicular-arbuscular endomycorrhizal fungi colonizing roots**. *Applied and Environmental Microbiology* 1992, **58**(1):291-295.

15. Lee J, Lee S, Young JPW: **Improved PCR primers for the detection and identification of arbuscular mycorrhizal fungi**. *FEMS Microbiology Ecology* 2008, **65**(2):339-349.

16. Sapkota R, Nicolaisen M: **High-throughput sequencing of nematode communities from total soil DNA extractions**. *BMC Ecology* 2015, **15**(1):3.

17. Martin M: **Cutadapt removes adapter sequences from high-throughput sequencing reads**. *EMBnetjournal; Vol 17, No 1: Next Generation Sequencing Data Analysis* 2011.

18. Callahan BJ, McMurdie PJ, Rosen MJ, Han AW, Johnson AJA, Holmes SP: **DADA2: High-resolution sample inference from Illumina amplicon data**. *Nat Methods* 2016, **13**(7):581-583.

19. Rognes T, Flouri T, Nichols B, Quince C, Mahé F: **VSEARCH: a versatile open source tool for metagenomics**. *PeerJ* 2016, **4**:e2584.

20. R Core team: **R: a language and environment for statistical computing. R Foundation for Statistical Computing, Vienna, Austria**. In*.*; 2015.

21. Abarenkov K, Henrik Nilsson R, Larsson K-H, Alexander IJ, Eberhardt U, Erland S, Høiland K, Kjøller R, Larsson E, Pennanen T *et al*: **The UNITE database for molecular identification of fungi – recent updates and future perspectives**. *New Phytol* 2010, **186**(2):281-285.

22. Öpik M, Vanatoa A, Vanatoa E, Moora M, Davison J, Kalwij JM, Reier Ü, Zobel M: **The online database MaarjAM reveals global and ecosystemic distribution patterns in arbuscular mycorrhizal fungi (Glomeromycota)**. *New Phytol* 2010, **188**(1):223-241.

23. Frøslev TG, Kjøller R, Bruun HH, Ejrnæs R, Brunbjerg AK, Pietroni C, Hansen AJ: **Algorithm for post-clustering curation of DNA amplicon data yields reliable biodiversity estimates**. *Nature Communications* 2017, **8**(1):1188.

24. McMurdie PJ, Holmes S: **phyloseq: An R package for reproducible interactive analysis and graphics of microbiome census data**. *PLoS One* 2013, **8**(4):e61217.
